# Association between intraoperative hypotension and myocardial injury after major open abdominal surgery

**DOI:** 10.3389/fmed.2026.1806976

**Published:** 2026-05-05

**Authors:** Haiying Ji, Ran Xia, Xingyu Tong, Shunan Lv, Wei Wang, Muqiao Cheng, Yi Zheng, Xueyin Shi, Chengmi Zhang

**Affiliations:** 1 Department of Anesthesiology and Critical Care Medicine, Xinhua Hospital Affiliated to Shanghai Jiaotong University School of Medicine, Shanghai, China; 2 Department of Anesthesiology, Qingpu Branch of Zhongshan Hospital Affiliated to Fudan University, Shanghai, China

**Keywords:** intraoperative hypotension, mean arterial pressure, myocardial injury after noncardiac surgery, open abdominal surgery, postoperative complications

## Abstract

**Background:**

Myocardial injury after non-cardiac surgery (MINS) contributes to mortality after major open abdominal surgery. While intraoperative hypotension (IOH) is a proposed cause, its impact—defined using comprehensive thresholds—in this specific high-risk cohort remains underexplored. This study aimed to determine the association between the severity and duration of IOH and MINS.

**Methods:**

We assessed patients undergoing open abdominal surgery with invasive blood pressure monitoring in a single-center retrospective cohort study conducted between June 2016 and July 2019. The primary exposure, IOH, was defined using three absolute and three relative thresholds of mean arterial pressure (MAP), according to established literature and clinical consensus. The primary outcome was MINS (serum Troponin T (TnT) ≥ 0.03 ng/mL within 30 days after surgery). A multivariable logistic regression analysis was applied to model the exposure–outcome relationship, adjusting for potential clinical confounders.

**Results:**

Of the 8,281 patients screened, 1,461 met the inclusion criteria. MINS occurred in 175 patients (12.0%). MAP below three absolute thresholds (≤65, ≤60, and ≤55 mmHg) and three thresholds relative to the preinduction MAP (decreases of >30, >40, and >50% from baseline) were independently associated with MINS. For MAP ≤60 mmHg, the odds of MINS increased with longer durations of hypotension, rising from 1.54 (95% CI 0.98–2.44) for 1–5 min to 3.81 (95% CI 2.20–6.61) for ≥21 min. At the longest duration, lower MAP thresholds were associated with higher risk (OR 3.66 for ≤65 mmHg; 3.81 for ≤60 mmHg; and 8.42 for ≤55 mmHg). Similar associations were observed for relative MAP reductions.

**Conclusion:**

In major open abdominal surgery patients, IOH, either based on absolute and relative thresholds, is independently and cumulatively associated with MINS. Associations based on absolute MAP thresholds appeared more consistent, whereas those based on relative reductions showed greater variability.

## 1 Introduction

Myocardial injury after non-cardiac surgery (MINS) is a common complication following moderate-to-high risk surgeries. It is frequently asymptomatic and is significantly associated with increased short- and long-term mortality (1). A major limitation in the generalizability of intraoperative hypotension (IOH) research is the substantial heterogeneity across surgical procedures, which differ markedly in physiological stress and underlying mechanisms of hypotension (2). Notably, patients undergoing major open abdominal surgery represent a distinct and high-risk population in whom the relationship between IOH and MINS has not been well-characterized. To address this gap, the present study specifically focuses on this surgical population.

This patient population is at a high risk for perioperative cardiovascular events due to several factors: (1) large surgical wounds and prolonged exposure leading to significant fluid evaporation and third-space fluid sequestration, resulting in effective circulatory volume depletion; (2) a pronounced systemic inflammatory response that can cause vasodilation and myocardial suppression; (3) deep anesthesia and muscle relaxation requirements that synergistically exacerbate circulatory depression; (4) extensive surgical incisions and high postoperative pain levels, increasing physiological stress; (5) the frequent presence of advanced age and severe comorbidities among patients; and (6) a relatively high incidence of emergency surgery, often leading to suboptimal preoperative preparation (3, 4). These characteristics make it particularly important to investigate the relationship between IOH and postoperative myocardial injury in this population.

Currently, there is no international consensus on defining IOH (5). Numerous studies have explored the associations between different blood pressure components—such as systolic, diastolic, and mean arterial pressure (MAP)—and postoperative myocardial injury; however, their conclusions remain inconsistent (6–8). Complex, time-varying biological signals are often best characterized by their central tendency, and MAP, as a time-integrated measure of the arterial waveform, effectively captures the central tendency of this complex, fluctuating signal and demonstrates a consistent correlation with aortic pressure (9–11). Therefore, six MAP thresholds were defined based on a review of the published literature: Three absolute thresholds (≤65, ≤60, and ≤55 mmHg) and three relative thresholds (decreases of >30, >40, and >50% from baseline) (12).

Based on this background, this study uses a retrospective cohort of patients who underwent major open abdominal surgery to determine whether IOH (defined by various MAP thresholds and durations) is independently associated with the risk of MINS, with the goal of informing tailored intraoperative blood pressure management strategies in this specific clinical scenario to ultimately improve patient outcomes.

## 2 Materials and methods

### 2.1 Study design and patients

This article adheres to the Strengthening the Reporting of Observational Studies in Epidemiology (STROBE) guidelines. We conducted a retrospective cohort study involving all patients scheduled for at least an overnight admission to the postoperative unit between June 2016 and July 2019. These patients underwent major open abdominal surgery (e.g., major gastrointestinal resection or hepatectomy), required general anesthesia with an expected duration of ≥2 h, and had invasive arterial pressure monitoring throughout the procedure. Patients who underwent laparoscopic surgery that was converted to open surgery were also included. We excluded patients who had surgeries with an estimated duration of less than 2 h, such as cholecystectomy, appendectomy, abdominal hernia repair, and abdominal mass resection. Patients with a preoperative serum troponin T (TnT) level ≥0.03 ng/mL and those without intraoperative arterial blood pressure data were also excluded.

### 2.2 Data collection

Demographic data and perioperative characteristics were obtained from electronic medical records, including age, sex, drinking and smoking status, comorbidities (such as hypertension, anemia, and diabetes), and preoperative laboratory test results (including hemoglobin levels and serum TnT). Anemia was defined according to the WHO criteria as a preoperative hemoglobin level <130 g/L in men and <120 g/L in women. Patients were classified based on the American Society of Anesthesiologists (ASA) physical status classification. Intraoperative data were obtained by reviewing anesthetic charts and included details such as surgical type (emergency surgery or elective surgery), abdominal surgical site, surgical duration, intraoperative blood loss, blood transfusion volume (including red blood cells and plasma), and intraoperative blood pressure and heart rate measurements. Surgical sites primarily included common general surgical locations such as the stomach, liver, bile duct, spleen, gallbladder, pancreas, small intestine, colon, and retroperitoneum, as well as multisite procedures (≥2 sites). The duration of surgery was defined as the time from the appearance of the first end-tidal carbon dioxide following anesthesia induction until the surgical incision was sutured. Postoperative variables such as serial TnT measurements and length of hospital stay were also obtained from the electronic medical record system.

### 2.3 Variable definitions and outcomes

Intraoperative heart rate and blood pressure data were recorded using the DoCare anesthesia information management system (MedicalSystem, China) at 1-min intervals. Data collection commenced with the first detection of end-tidal carbon dioxide after anesthesia induction and continued until the end of surgery. The raw invasive blood pressure data underwent a rigorous, multi-stage manual cleaning process prior to analysis. This process was conducted to exclude known sources of artifacts and errors. Specifically, data points that corresponded to periods of transducer flushing, leveling, and zeroing were first removed. Subsequently, the dataset was screened for physiologically implausible values and unannotated abrupt changes. Records were excluded if they met any of the following criteria: (1) systolic blood pressure ≥300 mmHg or ≤20 mmHg, (2) diastolic blood pressure ≥225 mmHg or ≤5 mmHg, or (3) adjacent values exhibiting a sudden change greater than 30 mmHg without a corresponding clinical annotation to justify the shift (13). In this study, we established six *a priori*-established IOH thresholds based on clinical practice and empirical evidence to ensure methodological rigor and comparability: three absolute MAP thresholds (≤65, ≤60, and ≤55 mmHg) and three relative MAP thresholds (decreases of >30, >40, and >50% from baseline) (9, 14). We calculated the cumulative duration for each patient as the total time (in min) during which MAP remained below a predefined threshold during surgery. Relative MAP thresholds were derived using a baseline value, which was computed as the average of all blood pressure recordings obtained in the operating room prior to the induction of anesthesia. Previous research indicates that the risk increases rapidly for each min spent below the threshold during the first 10 min, with a slower rate of increase thereafter (11). Therefore, the duration of IOH was categorized into five groups (<1, 1 to 5, 6 to 10, 11 to 20, and >20 min). Following the VISION study criteria, the primary outcome—postoperative myocardial injury—was defined as an elevated serum TnT level (≥0.03 ng/mL) measured within 30 days after surgery. TnT elevations were independently adjudicated as MINS through a structured review of electronic medical records. For each case, perioperative documentation, progress notes, discharge diagnoses, ECGs, available imaging data, and records of major complications were reviewed to identify alternative non-ischemic explanations. TnT elevations were not classified as MINS if a clear non-ischemic cause (such as sepsis, pulmonary embolism, severe arrhythmia, cardiopulmonary resuscitation, or myocarditis) was documented (15, 16). TnT levels were measured on postoperative days 1, 2, 3, 5, 7, 15, and 28, and the highest recorded TnT value was used for each patient.

### 2.4 Statistical analysis

Data were analyzed using SPSS Statistics version 26.0 (IBM, NY, United States) and R version 4.4.1 (R Project for Statistical Computing, Vienna, Austria). Continuous variables were assessed for normality using the Kolmogorov–Smirnov test. Normally distributed data were presented as mean ± standard deviation and compared using Student’s *t*-test; otherwise, data were presented as median (interquartile range) and compared using the Mann–Whitney U test. Categorical variables were reported as absolute numbers (percentages) and analyzed using the chi-squared test or Fisher’s exact test, as appropriate. A two-sided *p* < 0.05 was considered statistically significant.

A univariable logistic regression analysis was performed to assess the crude associations between the primary exposure (cumulative duration under absolute and relative MAP thresholds), key confounding variables (such as age, ASA physical status classification, emergency surgery, and preoperative comorbidities), and the outcome of MINS.

A multivariable logistic regression model was constructed to assess the independent association between IOH and MINS. The model was adjusted for prespecified potential confounders identified through univariable analysis, including continuous variables such as Age, operative time, red blood cell transfusion volume, plasma transfusion volume, and blood loss, and dichotomous variables such as ASA grade, hypertension, anemia, diabetes mellitus, emergency surgery, gastric surgery, and multisite surgery. The results of logistic regression analyses were presented as odds ratios (ORs) with 95% confidence intervals (CIs).

Several sensitivity analyses were conducted to assess the robustness of our findings. First, we repeated the primary multivariable logistic regression analysis after excluding all patients who underwent emergency surgery. Second, we excluded patients aged over 75 years and repeated the primary analysis to examine whether the results were primarily driven by advanced age-related risks. Third, we excluded patients who underwent multisite surgery (involving two or more abdominal organs) to isolate the effect of IOH in patients undergoing standardized, single-site open abdominal procedures. Fourth, to isolate the independent effect of moderate hypotension from profound hypotension, we conducted a sensitivity analysis focusing on specific MAP bands (17). This involved repeating the multivariable analysis for patients whose blood pressure levels fell within intermediate ranges (e.g., 55–60 mmHg, 60–65 mmHg, or relative decreases of 30–40% and 40–50% from baseline), thereby ensuring that the risk attributed to higher thresholds was not confounded by concurrent exposure to lower blood pressure levels.

Given the retrospective design, the sample size was determined by the number of eligible patients available during the study period, and no formal *a priori* sample size calculation was performed. This approach is consistent with observational studies based on existing clinical data. To ensure the robustness of the multivariable analysis, we evaluated the events-per-variable (EPV) ratio. With 175 myocardial injury events, the EPV ratio exceeded commonly recommended thresholds, supporting the stability and reliability of the regression models.

## 3 Results

**Figure 1:**
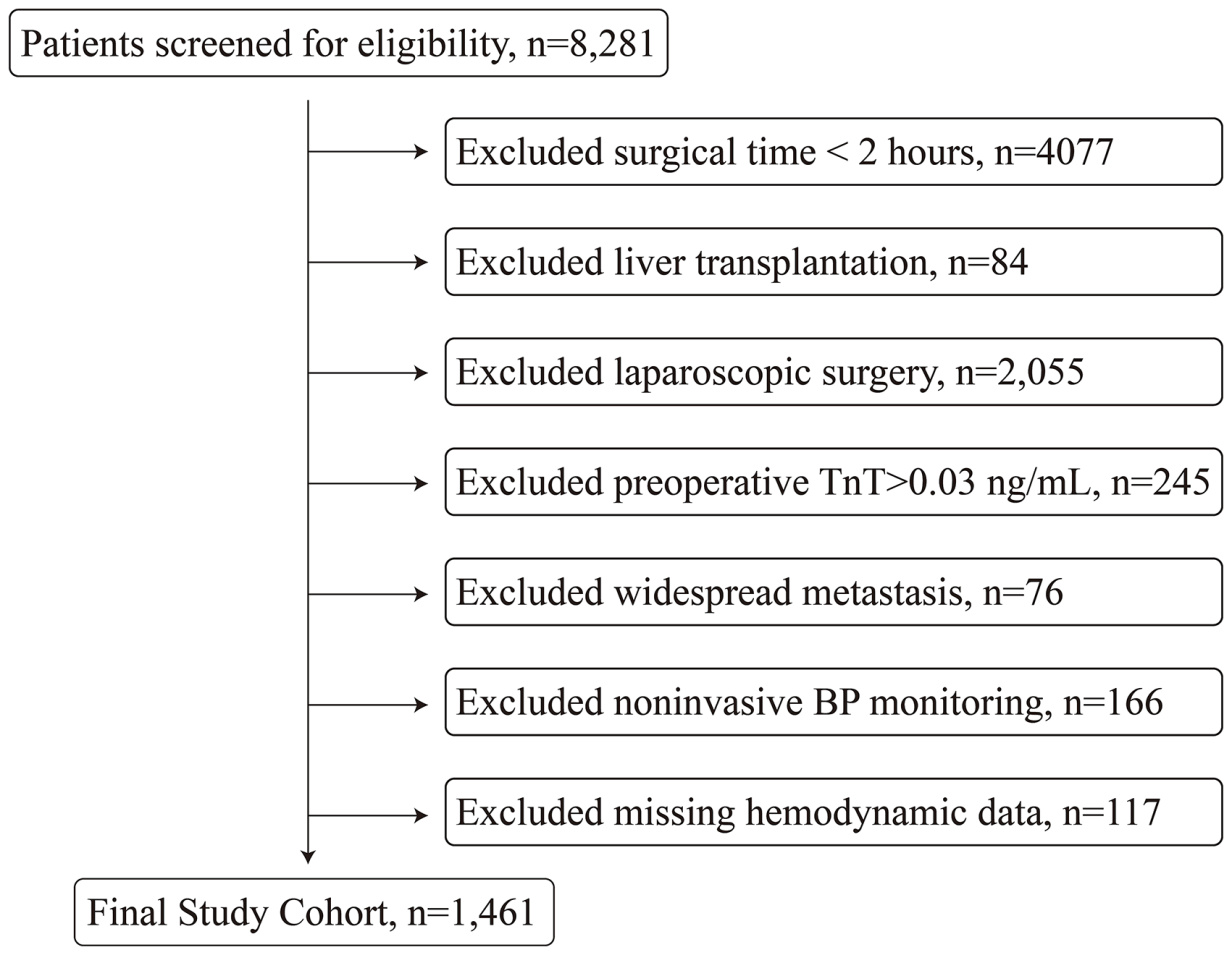
Flowchart of patient inclusion.

In total, 1,461 patients were eligible for inclusion (Figure 1). MINS occurred in 175 (12.0%) patients. Table 1 compares the characteristics of patients with and without MINS. In the overall cohort, the median age was 63 years and 65.8% of patients were men. The most prevalent comorbidity was hypertension (31.1%), and the most common type of surgery was gastric surgery (38.4%), followed by multisite surgery (19.4%). Compared to patients without myocardial injury, those in the injury group were older, had higher ASA physical status classifications and more comorbidities, underwent more emergency and multisite surgeries, and experienced longer operative times, greater blood loss, and higher transfusion requirements. However, there was no significant difference in the postoperative length of hospital stay between the two groups (Table 1).

**Table 1:** Characteristics of the cohort and incidence of MINS within 30 days after surgery.

Characteristics	Total, *n* = 1,461	Without MINS, *n* = 1,285	With MINS, *n* = 175	*p*
Demographics				
Age (yr)	63(56, 70)	63(54, 69)	70 (63,78)	<0.001
Sex (%)				0.851
Male	961 (65.8)	847 (65.9)	114 (65.1)	
Female	500 (34.2)	439 (34.1)	61 (34.9)	
Drinking (%)	122 (8.4)	113 (8.8)	9 (5.1)	0.102
Smoking (%)	210 (14.4)	189 (14.7)	21 (12.0)	0.340
Preoperative characteristics				
ASA physical status classification (%)				<0.001
1	3 (0.2)	3 (0.2)	0 (0.0)	
2	835 (57.2)	768 (59.7)	67 (38.3)	
3	595 (40.7)	500 (38.9)	95 (54.3)	
4	28 (1.9)	15 (1.2)	13 (7.4)	
Comorbidities (%)				
Anemia	254 (17.4)	207 (16.1)	47 (26.9)	<0.001
Hypertension	455 (31.1)	383 (29.8)	72 (41.1)	0.002
Diabetes	178 (12.2)	146 (11.4)	32 (18.3)	0.009
Basic MAP (mmHg)	96.0 (89.3, 105.0)	96.0 (89.0, 105.0)	98.0 (90.0, 107.0)	0.173
Surgery characteristics				
Emergency surgery (%)	80 (5.5)	55 (4.3)	25 (14.3)	<0.001
Open abdominal surgical site (%)				
Gastric surgery	561 (38.4)	510 (39.7)	51 (29.1)	0.007
Liver surgery	125 (8.6)	111 (8.6)	14 (8.0)	0.779
Bile duct surgery	12 (0.8)	10 (0.8)	2 (1.1)	0.955
Gallbladder surgery	17 (1.2)	15 (1.2)	2 (1.1)	1.000
Splenic surgery	17 (1.2)	15 (1.2)	2 (1.1)	1.000
Pancreatic operation	156 (10.7)	133 (10.3)	23 (13.1)	0.260
Small intestine surgery	42 (2.9)	38 (3.0)	4 (2.3)	0.619
Colorectal surgery	215 (14.7)	189 (14.7)	26 (14.9)	0.955
Retroperitoneal surgery	32 (2.2)	30 (2.3)	2 (1.1)	0.463
Multisite surgery	284 (19.4)	235 (18.3)	49 (28.0)	0.002
Intraoperative characteristics				
Duration of surgery (min)	185 (150, 240)	180 (150, 240)	210 (150, 250)	0.016
Red blood cell transfusion (U)	0 (0, 2)	0 (0, 0)	0 (0, 3)	<0.001
Plasma transfusion (ml)	0 (0, 0)	0 (0, 0)	0 (0, 400)	<0.001
Blood loss (ml)	200 (100, 300)	200 (100, 300)	250 (100, 550)	<0.001
Postoperative characteristics				
Length of hospital stay (days)	14 (11, 20)	14 (11, 20)	13 (11, 19)	0.548

MINS, myocardial injury after non-cardiac surgery; MAP, mean arterial pressure; ASA, American Society of Anesthesiologists.

We found that older age, higher ASA physical status classification, preexisting comorbidities (including anemia, hypertension, and diabetes mellitus), emergency surgery, gastric surgery, multisite surgery, prolonged surgery duration, increased blood product transfusion, and greater intraoperative blood loss were also associated with MINS in the univariable analysis (Supplementary Table S1). Therefore, these variables were considered potential confounders and were subsequently included in the multivariable regression model to adjust for confounding.

The univariable associations between thresholds and durations of IOH, defined using both absolute and relative MAP thresholds, and the risk of MINS are presented in Supplementary Table S2. For absolute MAP thresholds, longer hypotension durations were associated with a higher likelihood of myocardial injury. At MAP ≤65 mmHg, the odds of injury increased from 1.92 (95% CI 1.09–3.37) for 1–5 min to 4.85 (95% CI 2.93–8.04) for durations ≥21 min, with the highest odds observed for MAP ≤55 mmHg lasting for ≥21 min (OR 10.59, 95% CI 5.15–21.75). Similar patterns were observed when hypotension was defined using relative MAP reductions. Overall, the univariable analysis demonstrated that more severe hypotension and longer cumulative durations of exposure, whether defined using absolute or relative thresholds, were associated with increased odds of MINS.

**Figure 2:**
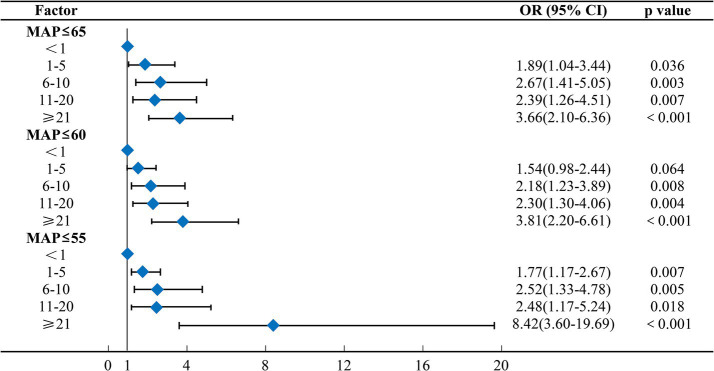
Multivariable logistic regression analysis of the association between intraoperative hypotension (defined using absolute thresholds) and postoperative myocardial injury. OR, odds ratio; CI, confidence interval; MAP, mean arterial pressure.

**Figure 3:**
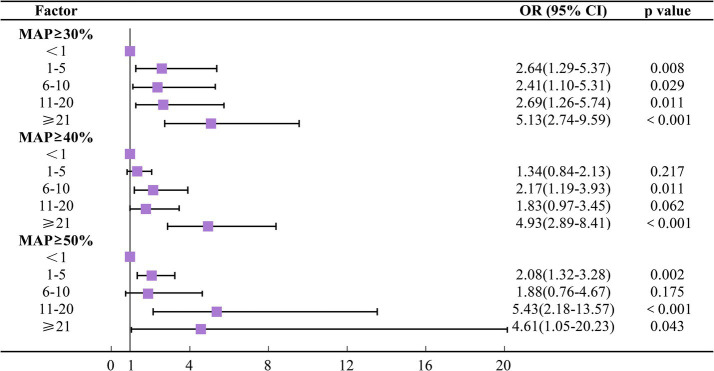
Multivariable logistic regression analysis of the association between intraoperative hypotension (defined using relative thresholds) and postoperative myocardial injury. OR, odds ratio; CI, confidence interval; MAP, mean arterial pressure.

After adjusting for potential confounders, lower blood pressure thresholds and longer durations of IOH were generally associated with higher odds of MINS. Using absolute MAP thresholds, increasing durations of hypotension were associated with progressively higher odds of MINS. For example, at MAP ≤60 mmHg, compared to the reference group, hypotension lasting 1–5 min was associated with an OR of 1.54 (95% CI 0.98–2.44). This OR increased to 2.18 (95% CI 1.23–3.89) for 6–10 min, 2.30 (95% CI 1.30–4.06) for 11–20 min, and 3.81 (95% CI 2.20–6.61) for durations ≥21 min. When the longest duration category (≥21 min) was examined across thresholds, higher odds of MINS were observed at lower MAP cutoffs, with ORs of 3.66 for MAP ≤65 mmHg, 3.81 for MAP ≤60 mmHg, and 8.42 for MAP ≤55 mmHg (Figure 2). Similar overall patterns were observed when hypotension was defined using relative MAP reductions. For a ≥ 30% reduction in MAP, hypotension lasting 6–10 min (OR 2.41, 95% CI 1.10–5.31), 11–20 min (OR 2.69, 95% CI 1.26–5.74), and ≥21 min (OR 5.13, 95% CI 2.74–9.59) was associated with increased odds of MINS (Figure 3). In addition, the associations were not strictly monotonic in some exposure categories, particularly at the most severe relative thresholds (e.g., MAP reductions ≥50%). In addition, slightly higher ORs were observed for milder hypotension categories (MAP ≤65 mmHg or ≥30% reduction) compared to more severe types (MAP ≤60 mmHg or ≥40% reduction). These models are presented in Supplementary Tables S3, S4. In the fully adjusted models, increasing age, emergency surgery, and higher ASA physical status classification were independently associated with the risk of MINS. Restricted cubic spline analyses further demonstrated a continuous, non-linear association between the cumulative duration of intraoperative hypotension and the predicted probability of MINS across both absolute and relative MAP thresholds (Supplementary Figure S1).

To evaluate the robustness of the association between IOH and MINS, sensitivity analyses were conducted by sequentially excluding patients who underwent emergency surgery (*n* = 80; Supplementary Tables S5, S6), those aged 75 years (*n* = 193; Supplementary Tables S7, S8), and those receiving combined surgical procedures (*n* = 284; Supplementary Tables S9, S10).

Notably, when IOH was defined using absolute thresholds (MAP ≤65, ≤60, or ≤55 mmHg), the adjusted ORs for MINS demonstrated a pattern of modest numerical increase after excluding high-risk subgroups (Table 2). For instance, when the MAP was ≤65 mmHg for ≥21 min, the OR increased from 3.66 (95% CI, 2.10–6.36) in the primary analysis to 4.04 (95% CI, 2.20–7.44) after excluding patients who underwent emergency surgery, and the OR further increased to 4.21 (95% CI, 2.16–8.19) after excluding elderly patients. In contrast, when IOH was defined using relative thresholds (≥30%, ≥40%, or ≥50% decrease from baseline MAP), greater variability in effect estimates was observed (Table 2). For example, for a ≥40% reduction in MAP lasting 6–10 min, the estimated ORs increased modestly after excluding patients who underwent emergency surgery, those who received combined surgical procedures, and elderly patients aged over 75 years. In contrast, for the same magnitude of MAP reduction lasting ≥21 min, excluding these high-risk subgroups was associated with a slight decrease in the strength of the association between IOH and MINS. Overall, these sensitivity analyses demonstrated that the associations based on absolute MAP thresholds were comparatively robust across different patient exclusions. However, the associations based on relative MAP reductions showed less consistent directions and magnitudes of change across the sensitivity analyses.

**Table 2:** Comparison of odds ratios for myocardial injury after non-cardiac surgery across primary and sensitivity analyses.

Duration	≤65 mmHg	≤60 mmHg	≤55 mmHg	≥30% decrease	≥40% decrease	≥50% decrease
<1	Reference	Reference	Reference	Reference	Reference	Reference
1–5						
Risk 0	1.89 (1.04–3.44)	1.54 (0.98–2.44)	1.77 (1.17–2.67)	2.64 (1.29–4.37)	1.34 (0.84–2.13)	2.08 (1.32–3.28)
Risk 1	2.05 (1.07–3.92)	1.56 (0.96–2.54)	1.77 (1.17–2.67)	2.53 (1.18–5.44)	1.15 (0.69–1.90)	2.20 (1.37–3.52)
Risk 2	1.92 (1.06–3.49)	1.56 (0.98–2.46)	1.77 (1.17–2.67)	2.61 (1.28–5.31)	1.34 (0.84–2.12)	2.11 (1.34–3.32)
Risk 3	1.75 (0.83–3.69)	1.74 (1.01–2.99)	1.94 (1.21–3.12)	3.40 (1.45–7.99)	1.51 (0.87–2.60)	2.35 (1.40–3.92)
6–10						
Risk 0	2.67 (1.41–5.05)	2.18 (1.22–3.89)	2.52 (1.33–4.78)	2.41 (1.10–5.31)	2.17 (1.19–3.93)	1.88 (0.76–4.67)
Risk 1	2.85 (1.44–5.65)	2.23 (1.22–4.09)	2.52 (1.33–4.78)	2.27 (0.98–5.30)	2.28 (1.23–4.22)	1.91 (0.74–4.93)
Risk 2	2.70 (1.43–5.11)	2.23 (1.25–3.96)	2.58 (1.37–4.88)	2.40 (1.09–5.28)	2.21 (1.22–4.00)	1.94 (0.79–4.79)
Risk 3	4.96 (2.40–10.27)	3.13 (1.66–5.91)	4.05 (2.04–8.04)	3.38 (1.30–8.80)	2.60 (1.36–4.98)	2.95 (1.08–8.03)
11–20						
Risk 0	2.39 (1.26–4.51)	2.30 (1.30–4.06)	2.48 (1.17–5.24)	2.69 (1.26–5.74)	1.83 (0.97–3.45)	5.43 (2.18–13.57)
Risk 1	2.36 (1.17–4.74)	2.62 (1.44–4.77)	2.48 (1.17–5.24)	2.65 (1.18–5.95)	2.09 (1.10–3.98)	5.30 (2.11–13.34)
Risk 2	2.43 (1.29–4.58)	2.28 (1.29–4.03)	2.46 (1.16–5.21)	2.70 (1.27–5.74)	1.90 (1.01–3.59)	5.27 (2.10–13.23)
Risk 3	2.61 (1.21–5.64)	2.72 (1.43–5.17)	3.03 (1.28–7.16)	2.91 (1.12–7.55)	2.62 (1.26–5.43)	5.32 (2.06–13.72)
≥21						
Risk 0	3.66 (2.10–6.36)	3.81 (2.20–6.61)	8.42 (3.60–19.69)	5.13 (2.74–9.59)	4.93 (2.89–8.41)	4.61 (1.05–20.23)
Risk 1	4.04 (2.20–7.44)	4.02 (2.25–7.16)	8.42 (3.60–19.69)	5.04 (2.58–9.86)	4.81 (2.76–8.35)	7.86 (1.71–36.02)
Risk 2	3.70 (2.13–6.43)	3.86 (2.23–6.69)	8.33 (3.55–19.54)	5.14 (2.75–9.60)	4.92 (2.89–8.38)	4.48 (1.00–20.06)
Risk 3	4.21 (2.16–8.19)	4.01 (2.13–7.57)	7.11 (2.87–17.61)	6.02 (2.77–13.09)	4.87 (2.71–8.75)	1.29 (0.13–12.60)

Risk 0 represents the primary analysis. Risk 1 represents the analysis excluding participants who underwent emergency surgery. Risk 2 represents the analysis excluding participants who underwent multisite surgery. Risk 3 represents the analysis excluding older patients (age >75 years).

To further evaluate whether the observed associations between IOH and MINS were driven primarily by extreme blood pressure values, additional sensitivity analyses were performed using intermediate absolute and relative hypotension bands (Supplementary Table S11). Within the MAP 55–60 mmHg band, increasing durations of hypotension were associated with progressively higher odds of myocardial injury, with ORs of 1.89 (95% CI 1.24–2.87) for 1–5 min, 1.96 (95% CI 1.13–3.38) for 6–10 min, 2.28 (95% CI 1.22–4.27) for 11–20 min, and 3.54 (95% CI 1.73–7.23) for durations ≥21 min. Comparable findings were observed when hypotension was defined using intermediate relative MAP reductions. For MAP decreases of 30–40%, the odds of myocardial injury increased with longer durations of hypotension, reaching an OR of 4.77 (95% CI 2.62–8.68) for durations ≥21 min. These findings underscore the importance of preventing and promptly correcting moderate and severe hypotensive episodes during major open abdominal surgery.

## 4 Discussion

The results of this retrospective cohort study demonstrate that, among patients undergoing major open abdominal surgery, the risk of MINS is independently associated with both the severity and the cumulative duration of intraoperative hypotension. In this study, the primary exposure—intraoperative hypotension—was defined using a combination of three absolute and three relative MAP thresholds. This dual framework was adopted to capture both population-level risk using absolute thresholds and interindividual susceptibility using relative thresholds—an approach supported by prior evidence and clinical relevance (18).

Overall, our findings build upon previous studies by showing that this association is particularly evident in the high-risk context of major open abdominal surgery. In general, lower achieved blood pressure levels and longer cumulative durations of hypotension were associated with higher odds of MINS. The distinctive pathophysiological features of these procedures—including substantial fluid shifts, the need for deep anesthesia and neuromuscular blockade, and frequent perioperative inflammation and pain—may be associated with reduced tolerance to even modest reductions in perfusion pressure.

Although increasing severity and duration of hypotension were generally associated with a higher risk of MINS, the associations were not strictly monotonic across all exposure categories. This apparent inconsistency may reflect the limitations of categorical analyses in capturing a fundamentally continuous and non-linear relationship. Consistent with this interpretation, spline analyses demonstrated a continuous, non-linear increase in the likelihood of MINS with longer durations of hypotension, which may explain why categorical analyses do not always show a perfectly monotonic trend. More importantly, this observation underscores the complexity of the relationship between IOH and myocardial injury, which likely reflects a combination of measurement variability, exposure heterogeneity, limited sample sizes in extreme exposure strata, and the influence of clinical interventions that actively modify both the severity and duration of hypotension during surgery.

Notably, milder hypotension thresholds (e.g., MAP ≤65 mmHg or a ≥30% reduction from baseline) were associated with odds ratios that were comparable to, and in some strata slightly higher than, those observed for more severe hypotension thresholds. This finding may reflect differences in baseline risk profiles and exposure contexts across hypotension categories. Mild hypotension occurs more frequently in larger and more heterogeneous patient populations, including individuals with limited hemodynamic reserve, in whom even moderate reductions in blood pressure levels may be associated with an increased likelihood of myocardial injury. In contrast, patients who tolerate progression to more severe hypotension may, on average, represent a physiologically more resilient subgroup, thereby attenuating the relative effect estimates observed in these categories. Given the observational design of this study, residual confounding and selection bias cannot be fully excluded, and causal inferences should be made with caution. Prospective studies are therefore warranted to further clarify the causal role of intraoperative blood pressure management in the prevention of myocardial injury.

The present findings also provide additional insights into the relationship between intraoperative hypotension and myocardial injury after non-cardiac surgery. First, in this heterogeneous surgical population, associations based on absolute MAP thresholds appeared more consistent across sensitivity analyses compared to those based on relative MAP reductions. This observation suggests that absolute thresholds may better capture physiologically meaningful hypotensive burden across patients with diverse baseline blood pressure profiles, potentially offering greater practical value for real-time intraoperative blood pressure management. Second, the more pronounced non-monotonic patterns observed with relative MAP reductions highlight the heterogeneity of surgical patients. Although relative thresholds account for baseline blood pressure, they may also amplify physiological variability, leading to greater variability in associations with myocardial injury. Third, significant associations found within intermediate hypotension bands highlight the clinical relevance of brief and moderate hypotension, warranting heightened vigilance.

Recently, two high-quality randomized trials demonstrated that intensive or individualized blood pressure management provides no incremental benefit over a conventional strategy (targeting MAP ≥65 mmHg) in major abdominal surgery (19, 20). Our findings significantly extend this evidence base by revealing that relatively brief and moderate reductions in blood pressure levels (e.g., MAP 55–65 mmHg lasting 1–5 min) may be associated with a higher likelihood of myocardial injury, specifically after major open abdominal surgery. Consequently, our findings support the continued use of the conventional strategy (MAP ≥65 mmHg) and provide a potential pathophysiological explanation for its application in this high-risk cohort. Meanwhile, as described above, associations based on absolute MAP thresholds appeared more consistent, whereas those based on relative MAP reductions exhibited greater variability. This may partly explain why individualized, relative threshold-based strategies have not consistently improved outcomes in randomized trials, as these strategies may be more sensitive to patient heterogeneity.

van Waes et al. (14) also investigated the association between IOH and postoperative myocardial injury in a study of elderly patients undergoing vascular surgery. The reported incidence was higher than that observed in our study. This discrepancy is likely attributable to their study population, which primarily included older patients undergoing high-risk vascular surgery, in whom coronary artery disease was highly prevalent. Furthermore, they explored the relationship using the area under the curve for IOH and found that the results were comparable to those obtained using simpler metrics based on specific thresholds and cumulative duration. This consistency reinforces the practical utility and robustness of the straightforward hypotension metrics employed in the present study.

This study has several strengths. First, we confined our analysis to patients who underwent continuous invasive arterial pressure monitoring. This approach mitigates potential confounding that results from the inaccuracies and intermittent nature of non-invasive blood pressure measurements commonly used in previous studies. Second, as serial cardiac troponin measurements were integrated into the standard postoperative care protocol, this approach enhanced the generalizability of our findings while minimizing the risks of outcome misclassification and selection bias. Third, in addition to the duration of IOH below six thresholds, the analysis of intermediate MAP bands (e.g., 55–60 mmHg) provided compelling evidence that the risk associated with higher thresholds is independent of the time spent at lower levels. This methodological refinement moves beyond simplistic “threshold” models and offers a more granular understanding of IOH as a composite of severity and duration.

Several limitations of this study should be considered. First, although we adjusted for a comprehensive set of clinically relevant covariates in the multivariable models, residual confounding from unmeasured factors cannot be entirely excluded. In particular, this study did not investigate the specific triggers of intraoperative hypotension, such as acute hemorrhage. In addition, important perioperative management variables—including vasoactive medication use, anesthetic depth, and overall hemodynamic management strategies—were not analyzed in detail (21). Fluid balance, including potential fluid overload, was also not directly quantified using a standardized definition. The roles of these factors are complex and time-dependent. Some factors (e.g., excessive anesthetic depth) may be confounders if present before hypotension, while others (e.g., vasopressor use) may function as mediators in response to it. However, the retrospective design and lack of precise timing data prevented us from reliably distinguishing these roles. Although related variables such as intraoperative blood loss and transfusion were included in the analysis, they may only partially reflect volume status and fluid management. Therefore, residual confounding related to perioperative management and unmeasured physiological factors may persist. Moreover, the multiple comparisons performed across different hypotension thresholds and duration categories increase the risk of type I error. Consequently, our findings should be interpreted with caution, and they require validation in further prospective studies with more detailed and standardized perioperative data.

Second, IOH, defined as MAP <55 mmHg or a decrease >50% from baseline, occurred in only a few patients, which may reflect adequate intraoperative care but could have reduced the stability of our regression model for this exposure category. Third, it is recognized that pre-induction blood pressure tends to be higher than preoperative clinical measurements (22). This systematic elevation implies that relative thresholds, calculated from this higher baseline, might overestimate the true hypotensive burden, potentially resulting in a conservative bias that underestimates the true associations. Fourth, the study population was derived from a single academic center, which may affect the generalizability of our findings. However, the use of strict, objective criteria for both exposure (invasive MAP) and outcome (MINS based on serial troponin measurements) strengthens the internal validity of our conclusions. Finally, the exclusion of a substantial proportion of initially screened patients may introduce selection bias and may limit the generalizability of the findings. In particular, the study population was restricted to patients who underwent major open abdominal surgery with continuous invasive arterial pressure monitoring, and therefore, the results may not be directly applicable to lower-risk populations or those managed with non-invasive monitoring. This reflects an inherent trade-off between internal validity and external generalizability in observational studies of this nature.

The clinical implications of our findings are substantial. They highlight the importance of vigilant intraoperative blood pressure management in this high-risk surgical cohort. Ultimately, randomized controlled trials are needed to determine whether interventions aimed at minimizing both the severity and duration of IOH, guided by the thresholds identified here, are associated with a reduced incidence of MINS and improve overall patient outcomes after major abdominal surgery.

## 5 Conclusion

Among patients who underwent major open abdominal surgery, the severity and duration of IOH were independently associated with MINS. Even brief and moderate reductions in blood pressure levels were associated with an increased risk. Associations based on absolute MAP thresholds appeared more consistent, whereas those based on relative MAP reductions showed greater variability, which may partly reflect differences in baseline MAP estimation and patient heterogeneity. These findings support heightened vigilance toward hypotensive episodes during major open abdominal surgery and highlight the need for prospective studies to further define optimal blood pressure management strategies for mitigating the risk of myocardial injury.

## Data Availability

The raw data supporting the conclusions of this article will be made available by the authors, without undue reservation.
