# Supplementary material for: Association between intraoperative hypotension and myocardial injury after major open abdominal surgery
Source: Front Med (Lausanne). 2026 May 5;13:1806976. doi: 10.3389/fmed.2026.1806976 (PMC13183821; doi:10.3389/fmed.2026.1806976)
Supplement: Supplementary file 1 [file Supplementary_file_1.docx]

**Association Between Intraoperative Hypotension and Myocardial Injury After Major Open Abdominal Surgery**

Supplementary files

**Table S1.** Univariable analysis of the association between risk factors and postoperative myocardial injury.

**Table S2**. Univariable analysis of the association between intraoperative hypotension and postoperative myocardial injury.

**Table S3**. Multivariable logistic regression analysis of the association between intraoperative hypotension (defined using absolute thresholds) and postoperative myocardial injury.

**Table S4**. Multivariable logistic regression analysis of the association between intraoperative hypotension (defined using relative thresholds) and postoperative myocardial injury.

**Figure S1.** Restricted cubic spline showing the association between duration of intraoperative hypotension (absolute MAP thresholds: Panels A-C; relative MAP reductions: Panels D-F) and postoperative myocardial injury (MINS).

**Table S5**. Sensitivity analysis of the association between intraoperative hypotension (defined using absolute thresholds) and myocardial injury after excluding patients undergoing emergency surgery.

**Table S6**. Sensitivity analysis of the association between intraoperative hypotension (defined using relative thresholds) and myocardial injury after excluding patients undergoing emergency surgery.

**Table S7**. Sensitivity analysis of the association between intraoperative hypotension (defined using absolute thresholds) and myocardial injury after excluding elderly patients (age >75 years).

**Table S8**. Sensitivity analysis of the association between intraoperative hypotension (defined using relative thresholds) and myocardial injury after excluding elderly patients (age >75 years).

**Table S9**. Sensitivity analysis of the association between intraoperative hypotension (defined using absolute thresholds) and myocardial injury after excluding patients undergoing multisite surgery.

**Table S10**. Sensitivity analysis of the association between intraoperative hypotension (defined using relative thresholds) and myocardial injury after excluding patients undergoing multisite surgery.

**Table S11**. Sensitivity analysis for comparison of odds ratios of and postoperative myocardial injury across mean arterial pressure “bands”.

**Table S1**. Univariable analysis of the association between risk factors and postoperative myocardial injury.

| Risk factor | Univariable analysis |  |
| --- | --- | --- |
|  | Crude OR(95% CI) | p |
| Age (yr) | 1.07(1.05-1.09) | ＜0.001 |
| ASA status (%) | 2.52(1.89-3.35) | ＜0.001 |
| Anemia | 1.91(1.33-2.76) | 0.001 |
| Hypertension | 1.65(1.19-2.28) | 0.003 |
| Diabetes | 1.75(1.15-2.66) | 0.009 |
| Emergency surgery (%) | 3.73(2.26-6.16) | ＜0.001 |
| Gastric surgery | 0.63(0.44-0.88) | 0.008 |
| Multisite surgery | 1.74(1.22-2.49) | 0.003 |
| Duration of surgery (min) | 1.003(1.001-1.005) | 0.003 |
| Red blood cell (U) | 1.23(1.14-1.32) | ＜0.001 |
| Plasma (ml) | 1.002(1.001-1.002) | ＜0.001 |
| Blood loss (ml) | 1.001(1.000-1.001) | ＜0.001 |

OR, odds ratios; CI, confidence intervals; ASA, American Society of Anesthesiologists

**Table S2**. Univariable analysis of the association between intraoperative hypotension and postoperative myocardial injury.

| Variables |  | Incidence, (%) | Crude OR(95% CI) | p |
| --- | --- | --- | --- | --- |
| Cumulative time in the lowest MAP categories (min) | | | | |
| MAP≤65 mmHg | <1 (Reference) | 22(5.4) |  |  |
|  | 1-5 | 32(9.8) | 1.92(1.09-3.37) | 0.023 |
|  | 6-10 | 26(13.4) | 2.73(1.50-4.95) | 0.001 |
|  | 11-20 | 26(14.9) | 2.48(1.37-4.49) | 0.003 |
|  | ≥21 | 69(21.6) | 4.85(2.93-8.04) | ＜0.001 |
| MAP≤60 mmHg | <1 (Reference) | 49(7.2) |  |  |
|  | 1-5 | 42(11.1) | 1.62(1.05-2.50) | 0.029 |
|  | 6-10 | 23(16.7) | 2.58(1.52-4.41) | ＜0.001 |
|  | 11-20 | 24(17.1) | 2.67(1.58-4.53) | ＜0.001 |
|  | ≥21 | 37(29.8) | 5.49(3.39-8.90) | ＜0.001 |
| MAP≤55 mmHg | <1 (Reference) | 80(8.2) |  |  |
|  | 1-5 | 46(14.7) | 1.95(1.32-2.87) | 0.001 |
|  | 6-10 | 19(23.8) | 3.50(2.00-6.16) | ＜0.001 |
|  | 11-20 | 14(25.0) | 3.75(1.96-7.16) | ＜0.001 |
|  | ≥21 | 16(48.5) | 10.59(5.15-21.75) | ＜0.001 |
| Cumulative time in the lowest % MAP decrease categories (min) | | | | |
| MAP≥30% decrease | <1 (Reference) | 14(8.0) |  |  |
|  | 1-5 | 26(14.9) | 2.28(1.17-4.45) | 0.016 |
|  | 6-10 | 17(9.7) | 2.70(1.30-5.62) | 0.008 |
|  | 11-20 | 20(11.4) | 2.71(1.33-5.49) | 0.006 |
|  | ≥21 | 98(56.0) | 5.71(3.20-10.20) | ＜0.001 |
| MAP≥40% decrease | <1 (Reference) | 66(37.7) |  |  |
|  | 1-5 | 35(20.0) | 1.55(1.01-2.39) | 0.048 |
|  | 6-10 | 20(11.4) | 2.48(1.44-4.27) | 0.001 |
|  | 11-20 | 17(9.7) | 2.63(1.47-4.72) | 0.001 |
|  | ≥21 | 37(21.1) | 6.05(3.78-9.67) | ＜0.001 |
| MAP≥50% decrease | <1 (Reference) | 116(66.3) |  |  |
|  | 1-5 | 37(21.1) | 2.68(1.77-4.04) | ＜0.001 |
|  | 6-10 | 8(4.6) | 3.05(1.35-6.93) | 0.008 |
|  | 11-20 | 10(5.7) | 5.61(2.51-12.55) | ＜0.001 |
|  | ≥21 | 4(2.3) | 7.63(2.02-28.83) | 0.003 |

OR, odds ratios; CI, confidence intervals; MAP, mean arterial pressure

**Table S3**. Multivariable logistic regression analysis of the association between intraoperative hypotension (defined using absolute thresholds) and postoperative myocardial injury.

| Risk factor | Adjusted OR(95% CI) | | |
| --- | --- | --- | --- |
|  | MAP≤65 mmHg | MAP≤60 mmHg | MAP≤55 mmHg |
| Age (yr) | 1.07(1.05-1.09) | 1.07(1.05-1.09) | 1.07(1.05-1.09) |
| ASA status (%) | 1.66(1.20-2.29) | 1.68(1.21-2.32) | 1.65(1.19-2.30) |
| Anemia | 1.35(0.88-2.07) | 1.32(0.86-2.03) | 1.28(0.83-1.98) |
| Hypertension | 1.17(0.81-1.69) | 1.16(0.80-1.67) | 1.17(0.81-1.70) |
| Diabetes | 1.28(0.80-2.04) | 1.25(0.78-1.99) | 1.23(0.77-1.97) |
| Emergency surgery (%) | 4.10(2.28-7.34) | 3.78(2.11-6.79) | 3.72(2.06-6.72) |
| Gastric surgery | 0.90(0.59-1.36) | 0.88(0.58-1.34) | 0.88(0.58-1.35) |
| Multisite surgery | 1.42(0.92-2.19) | 1.42(0.92-2.21) | 1.48(0.95-2.30) |
| Duration of surgery (min) | 1.003(1.000-1.005) | 1.002(1.000-1.005) | 1.003(1.000-1.005) |
| Red blood cell (U) | 1.10(0.94-1.29) | 1.09(0.93-1.28) | 1.09(0.93-1.28) |
| Plasma (ml) | 1.000(0.999-1.001) | 1.000(0.999-1.001) | 1.000(0.999-1.001) |
| Blood loss (ml) | 1.000(1.000-1.001) | 1.000(1.000-1.001) | 1.000(1.000-1.001) |
| Duration of Intraoperative Hypotension (min) | | | |
| <1 (Reference) |  |  |  |
| 1-5 | 1.89(1.04-3.44) | 1.54(0.98-2.44) | 1.77(1.17-2.67) |
| 6-10 | 2.67(1.41-5.05) | 2.18(1.22-3.89) | 2.52(1.33-4.78) |
| 11-20 | 2.39(1.26-4.51) | 2.30(1.30-4.06) | 2.48(1.17-5.24) |
| ≥21 | 3.66(2.10-6.36) | 3.81(2.20-6.61) | 8.42(3.60-19.69) |

OR, odds ratios; CI, confidence intervals; MAP, mean arterial pressure; ASA, American Society of Anesthesiologists.

The analysis was adjusted for the potential confounding variables: age, ASA status, anemia, hypertension, diabetes, emergency surgery, gastric surgery, multisite surgery, duration of surgery, red blood cell transfusion, plasma transfusion, and blood loss.

**Table S4**. Multivariable logistic regression analysis of the association between intraoperative hypotension (defined using relative thresholds) and postoperative myocardial injury.

| Risk factor | Adjusted OR(95% CI) | | |
| --- | --- | --- | --- |
|  | MAP≥30% decrease | MAP≥40% decrease | MAP≥50% decrease |
| Age (yr) | 1.07(1.05-1.09) | 1.07(1.05-1.09) | 1.07(1.05-1.09) |
| ASA status (%) | 1.68(1.21-2.32) | 1.64(1.18-2.27) | 1.60(1.16-2.22) |
| Anemia | 1.52(0.98-2.35) | 1.38(0.89-2.13) | 1.37(0.89-2.11) |
| Hypertension | 1.02(0.71-1.48) | 1.01(0.70-1.47) | 1.08(0.75-1.56) |
| Diabetes | 1.26(0.79-2.01) | 1.39(0.87-2.21) | 1.30(0.81-2.08) |
| Emergency surgery (%) | 4.21(2.34-7.57) | 4.15(2.30-7.48) | 4.21(2.35-7.52) |
| Gastric surgery | 0.89(0.59-1.36) | 0.98(0.64-1.49) | 0.92(0.60-1.39) |
| Multisite surgery | 1.43(0.92-2.22) | 1.44(0.92-2.24) | 1.48(0.96-2.30) |
| Duration of surgery (min) | 1.003(1.000-1.005) | 1.003(1.000-1.005) | 1.003(1.000-1.006) |
| Red blood cell (U) | 1.10(0.94-1.29) | 1.13(0.96-1.33) | 1.12(0.95-1.31) |
| Plasma (ml) | 1.000(0.999-1.001) | 1.000(0.998-1.001) | 1.000(0.999-1.001) |
| Blood loss (ml) | 1.000(1.000-1.001) | 1.000(1.000-1.001) | 1.000(1.000-1.001) |
| Duration of Intraoperative Hypotension (min) | | | |
| <1 (Reference) |  |  |  |
| 1-5 | 2.64(1.29-4.37) | 1.34(0.84-2.13) | 2.08(1.32-3.28) |
| 6-10 | 2.41(1.10-5.31) | 2.17(1.19-3.93) | 1.88(0.76-4.67) |
| 11-20 | 2.69(1.26-5.74) | 1.83(0.97-3.45) | 5.43(2.18-13.57) |
| ≥21 | 5.13(2.74-9.59) | 4.93(2.89-8.41) | 4.61(1.05-20.23) |

OR, odds ratios; CI, confidence intervals; MAP, mean arterial pressure; ASA, American Society of Anesthesiologists.

The analysis was adjusted for the potential confounding variables: age, ASA status, anemia, hypertension, diabetes, emergency surgery, gastric surgery, multisite surgery, duration of surgery, red blood cell transfusion, plasma transfusion, and blood loss.

**Figure S1.** Restricted cubic spline showing the association between duration of intraoperative hypotension (absolute MAP thresholds: Panels A-C; relative MAP reductions: Panels D-F) and postoperative myocardial injury (MINS). Solid lines represent adjusted predicted probabilities with 95% confidence intervals (shaded areas).


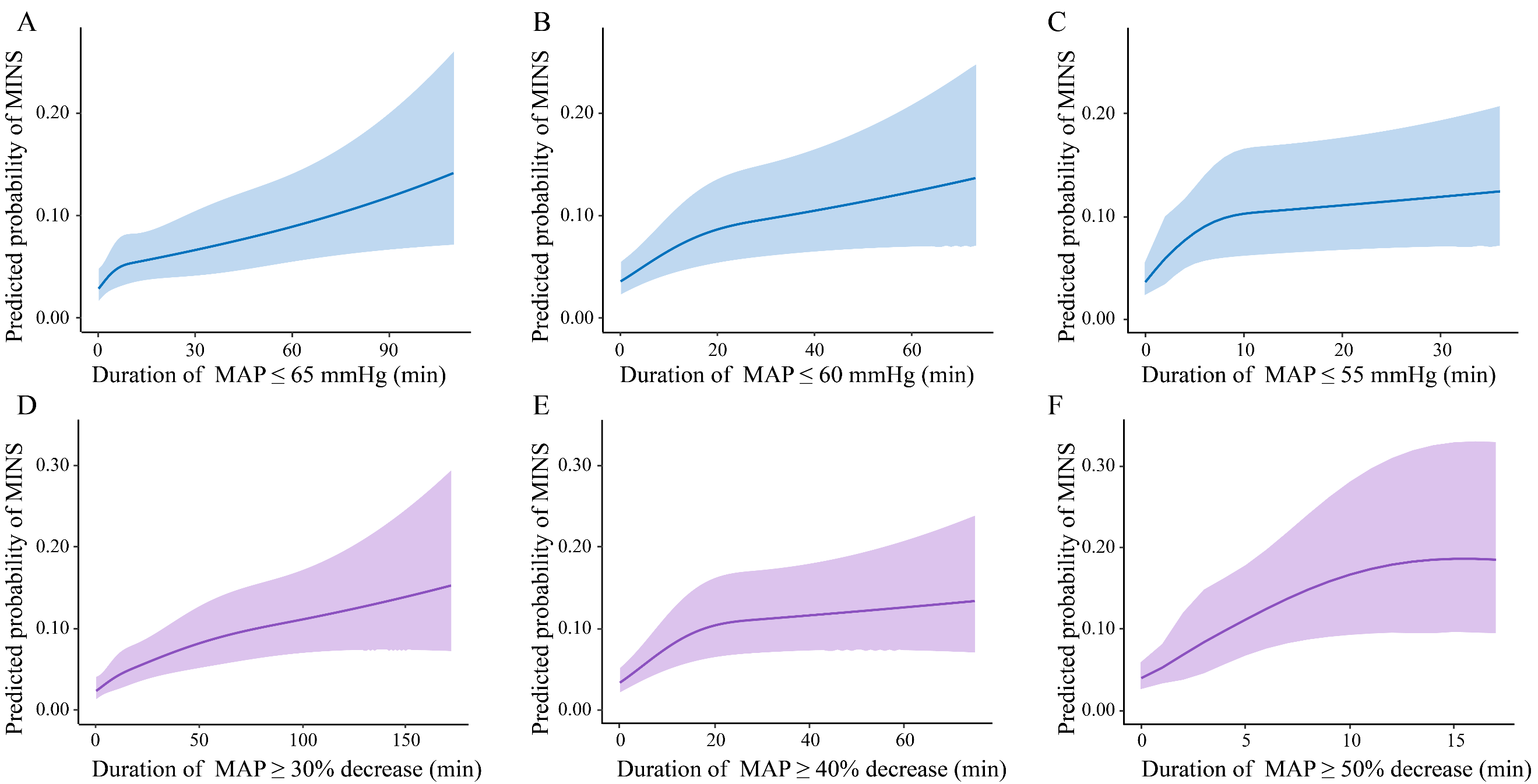


Predicted probabilities were derived from multivariable logistic regression models adjusted for age, ASA physical status, anemia, hypertension, diabetes, emergency surgery, gastric surgery, multisite surgery, duration of surgery, red blood cell transfusion, plasma transfusion, and intraoperative blood loss.

**Table S5**. Sensitivity analysis of the association between intraoperative hypotension (defined using absolute thresholds) and myocardial injury after excluding patients undergoing emergency surgery.

| Risk factor |  | Adjusted OR(95% CI) |  |
| --- | --- | --- | --- |
|  | MAP≤65 mmHg | MAP≤60 mmHg | MAP≤55 mmHg |
| Age (yr) | 1.07(1.04-1.09) | 1.07(1.05-1.09) | 1.07(1.05-1.09) |
| ASA status (%) | 1.49(1.06-2.10) | 1.53(1.08-2.16) | 1.51(1.06-2.15) |
| Anemia | 1.41(0.9-2.20) | 1.36(0.87-2.14) | 1.27(0.80-2.02) |
| Hypertension | 1.14(0.77-1.67) | 1.11(0.75-1.64) | 1.13(0.76-1.67) |
| Diabetes | 1.42(0.88-2.30) | 1.43(0.88-2.31) | 1.41(0.86-2.29) |
| Gastric surgery | 0.84(0.54-1.30) | 0.81(0.52-1.26) | 0.81(0.52-1.26) |
| Multisite surgery | 1.29(0.82-2.04) | 1.30(0.82-2.06) | 1.35(0.85-2.14) |
| Duration of surgery (min) | 1.002(0.999-1.005) | 1.002(0.999-1.005) | 1.002(0.999-1.005) |
| Red blood cell (U) | 1.108(0.944-1.301) | 1.100(0.934-1.295) | 1.110(0.941-1.309) |
| Plasma (ml) | 1.000(0.998-1.001) | 1.000(0.998-1.001) | 1.000(0.998-1.001) |
| Blood loss (ml) | 1.000(1.000-1.001) | 1.000(1.000-1.001) | 1.000(1.000-1.001) |
| Duration of Intraoperative Hypotension | | | |
| <1 (Reference) |  |  |  |
| 1-5 | 2.05(1.07-3.92) | 1.56(0.96-2.54) | 1.77(1.17-2.67) |
| 6-10 | 2.85(1.44-5.65) | 2.23(1.22-4.09) | 2.52(1.33-4.78) |
| 11-20 | 2.36(1.17-4.74) | 2.62(1.44-4.77) | 2.48(1.17-5.24) |
| ≥21 | 4.04(2.20-7.44) | 4.02(2.25-7.16) | 8.42(3.60-19.69) |

OR, odds ratios; CI, confidence intervals; MAP, mean arterial pressure; ASA, American Society of Anesthesiologists.

The analysis was adjusted for the potential confounding variables: age, ASA status, anemia, hypertension, diabetes, gastric surgery, multisite surgery, duration of surgery, red blood cell transfusion, plasma transfusion, and blood loss.

**Table S6**. Sensitivity analysis of the association between intraoperative hypotension (defined using relative thresholds) and myocardial injury after excluding patients undergoing emergency surgery.

| Risk factor | Adjusted OR(95% CI) | | |
| --- | --- | --- | --- |
|  | MAP≥30% decrease | MAP≥40% decrease | MAP≥50% decrease |
| Age (yr) | 1.07(1.05-1.09) | 1.07(1.05-1.09) | 1.07(1.05-1.09) |
| ASA status (%) | 1.47(1.04-2.08) | 1.48(1.05-2.10) | 1.41(0.99-1.99) |
| Anemia | 1.60(1.02-2.52) | 1.49(0.95-2.34) | 1.42(0.90-2.22) |
| Hypertension | 0.97(0.66-1.43) | 0.94(0.64-1.39) | 1.04(0.70-1.53) |
| Diabetes | 1.38(0.85-2.24) | 1.57(0.96-2.54) | 1.48(0.91-2.41) |
| Gastric surgery | 0.81(0.52-1.26) | 0.88(0.57-1.37) | 0.83(0.54-1.29) |
| Multisite surgery | 1.27(0.80-2.00) | 1.29(0.81-2.05) | 1.34(0.85-2.12) |
| Duration of surgery (min) | 1.002(1.000-1.005) | 1.002(1.000-1.005) | 1.003(1.000-1.005) |
| Red blood cell (U) | 1.113(0.946-1.309) | 1.133(0.959-1.340) | 1.131(0.958-1.335) |
| Plasma (ml) | 1.000(0.998-1.001) | 1.000(0.998-1.001) | 1.000(0.998-1.001) |
| Blood loss (ml) | 1.000(1.000-1.001) | 1.000(1.000-1.001) | 1.000(1.000-1.001) |
| Duration of Intraoperative Hypotension | | | |
| <1 (Reference) |  |  |  |
| 1-5 | 2.53(1.18-5.44) | 1.15(0.69-1.90) | 2.20(1.37-3.52) |
| 6-10 | 2.27(0.98-5.30) | 2.28(1.23-4.22) | 1.91(0.74-4.93) |
| 11-20 | 2.65(1.18-5.95) | 2.09(1.10-3.98) | 5.30(2.11-13.34) |
| ≥21 | 5.04(2.58-9.86) | 4.81(2.76-8.35) | 7.86(1.71-36.02) |

OR, odds ratios; CI, confidence intervals; MAP, mean arterial pressure; ASA, American Society of Anesthesiologists.

The analysis was adjusted for the potential confounding variables: age, ASA status, anemia, hypertension, diabetes, gastric surgery, multisite surgery, duration of surgery, red blood cell transfusion, plasma transfusion, and blood loss.

**Table S7**. Sensitivity analysis of the association between intraoperative hypotension (defined using absolute thresholds) and myocardial injury after excluding elderly patients (age >75 years).

| Risk factor | Adjusted OR(95% CI) | | |
| --- | --- | --- | --- |
|  | MAP≤65 mmHg | MAP≤60 mmHg | MAP≤55 mmHg |
| ASA status (%) | 2.25(1.55-3.25) | 2.21(1.53-3.20) | 2.20(1.52-3.20) |
| Anemia | 1.33(0.79-2.25) | 1.32(0.78-2.22) | 1.38(0.81-2.34) |
| Hypertension | 1.64(1.07-2.52) | 1.63(1.07-2.50) | 1.65(1.07-2.53) |
| Diabetes | 1.15(0.80-2.04) | 1.14(0.65-1.99) | 1.10(0.63-1.93) |
| Emergency surgery (%) | 2.50(1.24-5.04) | 2.31(1.14-4.67) | 2.16(1.05-4.46) |
| Gastric surgery | 0.92(0.56-1.51) | 0.95(0.58-1.56) | 0.90(0.55-1.48) |
| Multisite surgery | 1.41(0.87-2.30) | 1.47(0.90-2.39) | 1.45(0.88-2.36) |
| Duration of surgery (min) | 1.002(0.999-1.005) | 1.002(1.000-1.005) | 1.003(1.000-1.005) |
| Red blood cell (U) | 1.070(0.905-1.266) | 1.048(0.885-1.241) | 1.039(0.876-1.232) |
| Plasma (ml) | 1.000(0.998-1.001) | 1.000(0.998-1.001) | 1.000(0.999-1.001) |
| Blood loss (ml) | 1.000(1.000-1.001) | 1.000(1.000-1.001) | 1.000(1.000-1.001) |
| Duration of Intraoperative Hypotension | | | |
| <1 (Reference) |  |  |  |
| 1-5 | 1.75(0.83-3.69) | 1.74(1.01-2.99) | 1.94(1.21-3.12) |
| 6-10 | 4.96(2.40-10.27) | 3.13(1.66-5.91) | 4.05(2.04-8.04) |
| 11-20 | 2.61(1.21-5.64) | 2.72(1.43-5.17) | 3.03(1.28-7.16) |
| ≥21 | 4.21(2.16-8.19) | 4.01(2.13-7.57) | 7.11(2.87-17.61) |

OR, odds ratios; CI, confidence intervals; MAP, mean arterial pressure; ASA, American Society of Anesthesiologists.

The analysis was adjusted for the potential confounding variables: ASA status, anemia, hypertension, diabetes, emergency surgery, gastric surgery, multisite surgery, duration of surgery, red blood cell transfusion, plasma transfusion, and blood loss.

**Table S8**. Sensitivity analysis of the association between intraoperative hypotension (defined using relative thresholds) and myocardial injury after excluding elderly patients (age >75 years).

| Risk factor | Adjusted OR(95% CI) | | |
| --- | --- | --- | --- |
|  | MAP≥30% decrease | MAP≥40% decrease | MAP≥50% decrease |
| ASA status (%) | 2.18(1.50-3.16) | 2.17(1.50-3.15) | 2.20(1.52-3.18) |
| Anemia | 1.60(0.94-2.72) | 1.53(0.90-2.60) | 1.46(0.86-2.47) |
| Hypertension | 1.32(0.86-2.03) | 1.34(0.87-2.07) | 1.47(0.96-2.26) |
| Diabetes | 1.12(0.64-1.95) | 1.20(0.68-2.09) | 1.13(0.65-1.97) |
| Emergency surgery (%) | 2.88(1.44-5.78) | 2.69(1.33-5.44) | 2.81(1.41-5.62) |
| Gastric surgery | 0.92(0.56-1.50) | 0.99(0.60-1.62) | 0.99(0.60-1.62) |
| Multisite surgery | 1.46(0.90-2.38) | 1.41(0.86-2.31) | 1.48(0.91-2.42) |
| Duration of surgery (min) | 1.003(1.000-1.005) | 1.003(1.000-1.006) | 1.003(1.000-1.006) |
| Red blood cell (U) | 1.067(0.903-1.261) | 1.083(0.911-1.286) | 1.079(0.908-1.282) |
| Plasma (ml) | 1.000(0.999-1.001) | 1.000(0.998-1.001) | 1.000(0.998-1.001) |
| Blood loss (ml) | 1.000(1.000-1.001) | 1.000(1.000-1.001) | 1.000(1.000-1.001) |
| Duration of Intraoperative Hypotension | | | |
| <1 (Reference) |  |  |  |
| 1-5 | 3.40(1.45-7.99) | 1.51(0.87-2.60) | 2.35(1.40-3.92) |
| 6-10 | 3.38(1.30-8.80) | 2.60(1.36-4.98) | 2.95(1.08-8.03) |
| 11-20 | 2.91(1.12-7.55) | 2.62(1.26-5.43) | 5.32(2.06-13.72) |
| ≥21 | 6.02(2.77-13.09) | 4.87(2.71-8.75) | 1.29(0.13-12.60) |

OR, odds ratios; CI, confidence intervals; MAP, mean arterial pressure; ASA, American Society of Anesthesiologists.

The analysis was adjusted for the potential confounding variables: ASA status, anemia, hypertension, diabetes, emergency surgery, gastric surgery, multisite surgery, duration of surgery, red blood cell transfusion, plasma transfusion, and blood loss.

**Table S9**. Sensitivity analysis of the association between intraoperative hypotension (defined using absolute thresholds) and myocardial injury after excluding patients undergoing multisite surgery.

| Risk factor |  | Adjusted OR(95% CI) |  |
| --- | --- | --- | --- |
|  | MAP≤65 mmHg | MAP≤60 mmHg | MAP≤55 mmHg |
| Age (yr) | 1.07(1.05-1.09) | 1.07(1.05-1.09) | 1.07(1.05-1.09) |
| ASA status (%) | 1.67(1.21-2.30) | 1.68(1.22-2.32) | 1.65(1.19-2.29) |
| Anemia | 1.36(0.88-2.08) | 1.33(0.87-2.05) | 1.29(0.83-2.00) |
| Hypertension | 1.16(0.81-1.68) | 1.15(0.80-1.66) | 1.17(0.81-1.69) |
| Diabetes | 1.28(0.81-2.04) | 1.25(0.78-1.99) | 1.23(0.77-1.97) |
| Emergency surgery (%) | 3.95(2.21-7.05) | 3.64(2.04-6.51) | 3.55(1.98-6.38) |
| Gastric surgery | 0.80(0.54-1.17) | 0.78(0.53-1.14) | 0.77(0.52-1.14) |
| Duration of surgery (min) | 1.003(1.000-1.005) | 1.003(1.000-1.005) | 1.003(1.000-1.005) |
| Red blood cell (U) | 1.107(0.947-1.295) | 1.097(0.935-1.287) | 1.102(0.939-1.294) |
| Plasma (ml) | 1.000(0.999-1.001) | 1.000(0.999-1.001) | 1.000(0.999-1.001) |
| Blood loss (ml) | 1.000(1.000-1.001) | 1.000(1.000-1.001) | 1.000(1.000-1.001) |
| Duration of Intraoperative Hypotension | | | |
| <1 (Reference) |  |  |  |
| 1-5 | 1.92(1.06-3.49) | 1.56(0.98-2.46) | 1.77(1.17-2.67) |
| 6-10 | 2.70(1.43-5.11) | 2.23(1.25-3.96) | 2.58(1.37-4.88) |
| 11-20 | 2.43(1.29-4.58) | 2.28(1.29-4.03) | 2.46(1.16-5.21) |
| ≥21 | 3.70(2.13-6.43) | 3.86(2.23-6.69) | 8.33(3.55-19.54) |

OR, odds ratios; CI, confidence intervals; MAP, mean arterial pressure; ASA, American Society of Anesthesiologists.

The analysis was adjusted for the potential confounding variables: age, ASA status, anemia, hypertension, diabetes, emergency surgery, gastric surgery, duration of surgery, red blood cell transfusion, plasma transfusion, and blood loss.

**Table S10**. Sensitivity analysis of the association between intraoperative hypotension (defined using relative thresholds) and myocardial injury after excluding patients undergoing multisite surgery.

| Risk factor | Adjusted OR(95% CI) | | |
| --- | --- | --- | --- |
|  | MAP≥30% decrease | MAP≥40% decrease | MAP≥50% decrease |
| Age (yr) | 1.07(1.05-1.09) | 1.07(1.05-1.09) | 1.07(1.05-1.09) |
| ASA status (%) | 1.68(1.22-2.33) | 1.64(1.19-2.28) | 1.60(1.16-2.21) |
| Anemia | 1.53(0.99-2.37) | 1.38(0.89-2.14) | 1.38(0.90-2.13) |
| Hypertension | 1.02(0.70-1.47) | 1.01(0.69-1.46) | 1.08(0.75-1.55) |
| Diabetes | 1.26(0.79-2.00) | 1.38(0.87-2.21) | 1.30(0.81-2.07) |
| Emergency surgery (%) | 4.03(2.25-7.23) | 3.98(2.22-7.16) | 4.04(2.27-7.19) |
| Gastric surgery | 0.79(0.54-1.16) | 0.86(0.59-1.27) | 0.80(0.54-1.18) |
| Duration of surgery (min) | 1.003(1.000-1.005) | 1.003(1.000-1.005) | 1.003(1.001-1.006) |
| Red blood cell (U) | 1.109(0.947-1.300) | 1.136(0.965-1.337) | 1.127(0.959-1.324) |
| Plasma (ml) | 1.000(0.999-1.001) | 1.000(0.998-1.001) | 1.000(0.999-1.001) |
| Blood loss (ml) | 1.000(1.000-1.001) | 1.000(1.000-1.001) | 1.000(1.000-1.001) |
| Duration of Intraoperative Hypotension | | | |
| <1 (Reference) |  |  |  |
| 1-5 | 2.61(1.28-5.31) | 1.34(0.84-2.12) | 2.11(1.34-3.32) |
| 6-10 | 2.40(1.09-5.28) | 2.21(1.22-4.00) | 1.94(0.79-4.79) |
| 11-20 | 2.70(1.27-5.74) | 1.90(1.01-3.59) | 5.27(2.10-13.23) |
| ≥21 | 5.14(2.75-9.60) | 4.92(2.89-8.38) | 4.48(1.00-20.06) |

OR, odds ratios; CI, confidence intervals; MAP, mean arterial pressure; ASA, American Society of Anesthesiologists.

The analysis was adjusted for the potential confounding variables: age, ASA status, anemia, hypertension, diabetes, emergency surgery, gastric surgery, duration of surgery, red blood cell transfusion, plasma transfusion, and blood loss.

**Table S11**. Sensitivity analysis for comparison of odds ratios of and postoperative myocardial injury across mean arterial pressure “bands”.

| Risk factor | Adjusted OR(95% CI) | | | |
| --- | --- | --- | --- | --- |
|  | 55-60 mmHg | 60-65 mmHg | 30-40% decrease | 40-50% decrease |
| Age (yr) | 1.07(1.05-1.09) | 1.07(1.05-1.09) | 1.07(1.05-1.09) | 1.07(1.05-1.09) |
| ASA status (%) | 1.63(1.18-2.25) | 1.65(1.20-2.28) | 1.66(1.20-2.30) | 1.66(1.20-2.30) |
| Anemia | 1.36(0.89-2.09) | 1.33(0.86-2.04) | 1.52(0.98-2.36) | 1.44(0.93-2.23) |
| Hypertension | 1.17(0.81-1.68) | 1.13(0.78-1.63) | 1.02(0.71-1.47) | 1.07(0.74-1.54) |
| Diabetes | 1.26(0.79-2.01) | 1.32(0.83-2.10) | 1.25(0.78-1.99) | 1.35(0.85-2.16) |
| Emergency surgery (%) | 3.70(2.06-6.63) | 3.82(2.12-6.87) | 4.07(2.26-7.33) | 4.04(2.23-7.30) |
| Gastric surgery | 0.88(0.58-1.34) | 0.90(0.59-1.36) | 0.90(0.59-1.37) | 0.97(0.64-1.48) |
| Multisite surgery | 1.43(0.93-2.22) | 1.36(0.88-2.10) | 1.41(0.91-2.19) | 1.46(0.94-2.28) |
| Duration of surgery (min) | 1.003(1.000-1.005) | 1.003(1.000-1.005) | 1.003(1.000-1.005) | 1.003(1.000-1.005) |
| Red blood cell (U) | 1.097(0.935-1.287) | 1.128(0.962-1.323) | 1.104(0.942-1.293) | 1.132(0.961-1.333) |
| Plasma (ml) | 1.000(0.999-1.001) | 1.000(0.998-1.001) | 1.000(0.999-1.001) | 1.000(0.998-1.001) |
| Blood loss (ml) | 1.001(1.000-1.001) | 1.000(1.000-1.001) | 1.000(1.000-1.001) | 1.000(1.000-1.001) |
| Duration of Intraoperative Hypotension | | | | |
| <1 (Reference) |  |  |  |  |
| 1-5 | 1.89(1.24-2.87) | 1.61(0.97-2.67) | 2.38(1.20-4.71) | 1.66(1.07-2.60) |
| 6-10 | 1.96(1.13-3.38) | 1.84(1.04-3.26) | 2.39(1.16-4.96) | 2.82(1.61-4.95) |
| 11-20 | 2.28(1.22-4.27) | 1.68(0.92-3.07) | 2.66(1.31-5.39) | 1.35(0.64-2.84) |
| ≥21 | 3.54(1.73-7.23) | 3.88(2.20-6.86) | 4.77(2.62-8.68) | 4.99(2.83-8.81) |

OR, odds ratios; CI, confidence intervals; MAP, mean arterial pressure; ASA, American Society of Anesthesiologists.

The analysis was adjusted for the potential confounding variables: age, ASA status, anemia, hypertension, diabetes, emergency surgery, gastric surgery, multisite surgery, duration of surgery, red blood cell transfusion, plasma transfusion, and blood loss.
